# Preparation of Activated Carbon-Reinforced Composite Beads Based on MnO_2_/MCM-41@Fe_3_O_4_ and Calcium Alginate for Efficient Removal of Tetracycline in Aqueous Solutions

**DOI:** 10.3390/polym16081115

**Published:** 2024-04-16

**Authors:** Zhigong Zheng, Ronghui Shi, Xiaoping Zhang, Yonghao Ni, Hui Zhang

**Affiliations:** 1 College of Material Engineering, Fujian Agriculture and Forestry University, Fuzhou 350002, China; zhigongzheng@fjut.edu.cn; 2 School of Ecological Environment and Urban Construction, Fujian University of Technology, Fuzhou 350118, China; shironghui@fjut.edu.cn (R.S.); 17711834282@163.com (X.Z.); 3 Department of Chemical Engineering, University of New Brunswick, Fredericton, NB E3B 5A3, Canada

**Keywords:** MnO_2_/MCM-41@Fe_3_O_4_, COFAC, alginate, composite beads, catalyst, tetracycline

## Abstract

Tetracycline (TC) is a common antibiotic; when untreated TC enters the environment, it will cause a negative impact on the human body through the food chain. In the present study, MnO_2_/MCM-41@Fe_3_O_4_ (FeMnMCM) prepared using a hydrothermal and redox method and *Camellia oleifera* shell-activated carbon (COFAC) prepared through alkali activation were encapsulated using alginate (ALG) and calcium chloride as a cross-linking matrix to give the composite beads COFAC–FeMnMCM–ALG. The resultant COFAC–FeMnMCM–ALG composite beads were then carefully characterized, showing a high immobilization of MnO_2_/MCM-41@Fe_3_O_4_, with porous COFAC as an effective bioadsorbent for enriching the pollutants in the treated samples. These bead catalysts were subsequently applied to the oxidative degradation of TC in a Fenton oxidation system. Several parameters affecting the degradation were investigated, including the H_2_O_2_ concentration, catalyst dosage, initial TC concentration, and temperature. A very high catalytic activity towards the degradation of TC was demonstrated. The electron paramagnetic resonance (EPR) and quenching results showed that ·OH and ·O_2_^−^ were generated in the system, with ·OH as the main radical species. In addition, the COFAC–FeMnMCM–ALG catalyst exhibited excellent recyclability/reusability. We conclude that the as-prepared COFAC–FeMnMCM–ALG composite beads, which integrate MnO_2_ and Fe_3_O_4_ with bioadsorbents, provide a new idea for the design of catalysts for advanced oxidation processes (AOPs) and have great potential in the Fenton oxidation system to degrade toxic pollutants.

## 1. Introduction

Tetracycline (abbreviated TC) is a common antibiotic that has been widely used because of its affordability and broad-spectrum antibacterial and oral use [1]. The current sewage treatment system is unable to fully degrade the TC that is present in municipal wastewater. Thus, TC enters the receiving environment [2]. The World Health Organization defined a recommended maximum residue limit of 100 μg/kg (alone or combined) for TC. However, concentrations as high as 450 μg/L have already been detected in surface water [3]. TC will eventually accumulate in aquatic organisms (such as fish, shrimp, etc.) and enter the human body through the food chain, resulting in drug resistance [4,5], which leads to the development of so-called “superbugs” that no longer respond to current treatment modalities [6]. Consequently, the development of an effective method that can readily degrade TC is urgently needed.

At present, the main technologies for wastewater treatment include adsorption [7,8], membrane treatment [9], biodegradation [10], and advanced oxidation processes such as photolysis [11], ozonation [10,12], the Fenton oxidation process [13], and so on. The Fenton oxidation process is one of the most widely practiced processes using transition metal ions as catalysts [14]. Fe_3_O_4_ nanoparticles (MNPs) are excellent catalysts for the Fenton oxidation process due to their excellent oxidation performance [15,16]. The strong magnetic properties of the material may induce undesired nanoparticle aggregation, resulting in decreased catalytic performance [17]. Hence, in recent years, different methods have been studied to improve their stability, including the application of a coating layer to the surface of the MNPs. Mesoporous silica (MCM-41) can be considered a coating layer due to its high biocompatibility, non-toxicity, and easy surface modification [18]. Recently, magnetic nanostructures with mesoporous silica shells, such as Fe_3_O_4_-mSiO_2_ [19] and Fe_3_O_4_@MCM-41-SB/Pd [17], have been developed.

In recent years, hybrid oxides of transition elements and iron have emerged as potential catalysts for oxidative degradation due to their superior catalytic and magnetic properties [20]. Several studies have shown that transition metals play an important role in the Fenton reaction because they accelerate the valence change of Fe^3+^ to Fe^2+^ and generate more free radicals [21,22,23]. Among transition metal oxides, MnO_2_ has attracted considerable attention in pollution control applications due to its low cost, non-toxicity, and high chemical stability [20,24]. For example, Homa Ghasemi et al. [20] reported the decolorization of wastewater by the Fenton oxidation process, which was conducted with catalysts that were produced as Fe_3_O_4_/CuO MnO_2_-Fe_3_O_4_/CuO hybrids. The experimental results revealed that the introduction of MnO_2_ enhanced the rate of the decolorization reaction. In particular, MnO_2_-Fe_3_O_4_/CuO exhibited higher MB removal efficiency than Fe_3_O_4_/CuO.

However, the recovery of the powder after the catalytic test is difficult due to their small particle sizes, which can lead to secondary pollution. To overcome this problem, several attempts were proposed, in which encapsulation by a polymer matrix is known to be a promoter pathway [16]. Alginates, as natural polysaccharides produced by brown algae, are widely used as polymer matrices due to their high bioavailability and low cost. Nouri et al. [25] made a composite with calcium alginate and TiO_2_. The composite was then used for the adsorption and photocatalytic degradation of Basic Blue 41. Hachemaoui et al. [16] investigated the performance of MC@CA composite beads in the oxidation degradation of MB dye. MC@CA was made by encapsulating Fe_3_O_4_@MCM-41 with calcium alginate (CA) as a cross-linked matrix. The results showed that the beads were easily separable and functioned as exceptional heterogeneous Fenton catalysts.

Nevertheless, research has shown that unmodified MCM-41 has limited and low biosorption capacity due to the weak interaction between its inert silicate amorphous framework and organic pollutants [26]. Activated carbon is gaining more attention due to its high bioadsorption capacity and large specific surface area. Marrakchi et al. [27] introduced activated carbon into mesoporous MCM-41 to facilitate the synthesis of a functionalized composite encapsulated in cross-linked alginate hydrogel beads. A cross-linked ECAC/MCM-41/ALG hydrogel composite was synthesized for its highly efficient and enhanced biosorption capability towards synthetic Basic Blue (BB) dye and an emerging Bisphenol A (BPA) plasticizer.

By taking advantage of the properties associated with Fe_3_O_4_ and calcium alginate, we prepared a new class of composite beads that exhibit catalytic activities and easy separability, suitable for the catalytic oxidative degradation of TC. MCM-41 was used to coat the magnetite Fe_3_O_4_ to prevent the formation of aggregates. MnO_2_ was in situ deposited onto MCM-41@Fe_3_O_4_ to enhance the catalytic capacities. MnO_2_/MCM-41@Fe_3_O_4_ was then used as the core of the heterogeneous Fenton catalysts. COFAC with a high bioadsorption capacity and a large specific surface area was prepared from Camellia oleifera shells using a method previously reported by our group [28]. The COFAC was then incorporated into the catalyst to enhance the biosorption performance of the composite beads. Finally, the MnO_2_/MCM-41@Fe_3_O_4_ and COFAC were encapsulated/cross-linked using alginate and the addition of CaCl_2_. Finally, the COFAC–FeMnMCM–ALG composite beads were obtained by freeze-drying. These beads were then used for the catalytic degradation of tetracycline in an aqueous solution. Furthermore, the effects of various factors on TC degradation, such as the initial H_2_O_2_ concentration, catalyst amount, initial TC concentration, and reaction temperature, were investigated. The reuse and iron leaching of the COFAC–FeMnMCM–ALG composite beads for the removal of TC were also investigated. The active oxygen species of the system were determined through a radical quenching experiment and the detection results of the EPR spectrometer, and the degradation mechanism was discussed.

## 2. Materials and Methods

Potassium hydroxide (AR, ≥95%), sodium hydroxide (AR, ≥96%), ferric chloride hexahydrate (AR, ≥99%), ferrous sulfate heptahydrate (AR, ≥99%), and calcium chloride (AR, ≥96%) were all from Sinopharm Chemical Reagent Co., Ltd. (Shanghai, China). Cetyltrimethylammonium bromide (CTAB) (AR, ≥99%), tetraethylorthosilicate (TEOS) (AR, ≥98%), potassium permanganate (AR, ≥99%), sodium alginate (AR), tetracycline (AR, ≥98%), tert-butanol (TBA), and 5,5-dimethyl-1-pyrroline N-oxide (DMPO) were purchased from Aladdin (Shanghai, China). Deionized water was prepared by Milli-Q (18 MΩ). *Camellia oleifera* shells were collected from naturally dried fruits near Baiyan Mountain, Minqing District, Fuzhou City, China.

### 2.1. Preparation of Camellia oleifera Shell-Activated Carbon (COFAC)

The preparation of *Camellia oleifera* shell-activated carbon (COFAC) followed the procedure described in a previous work [28]. The shells of *Camellia oleifera* were cleaned and dried at 105 °C for 10 h. Subsequently, they were crushed and sieved to collect those passing through 100 meshes. These fine particles were carbonized under a N_2_ atmosphere at 400 °C for 1 h. The biochar was mixed with KOH in a 1:3 (*w*/*w*) ratio and activated at 650 °C for 1 h under a N_2_ atmosphere. After washing and drying at 100 °C for 24 h, the COFAC sample was obtained, which was stored in a desiccator until further use.

### 2.2. Synthesis of MnO_2_/MCM-41@Fe_3_O_4_ Composites

Fe_3_O_4_ magnetic nanoparticles (MNPs) were prepared using the precipitation method [29,30]. Firstly, 7.5 g of FeCl_3_·6H_2_O and 3.1 g of FeCl_2_·4H_2_O were dissolved in 160 mL of deionized water separately and dispersed through ultrasonic treatment for 30 min under a N_2_ atmosphere at 70 °C. The solution was mixed and adjusted to pH = 9–10 with 1.0 mol/L NaOH solution, stirring for an additional 2 h. The precipitated magnetite was washed 5 times to neutralize it, separated by a magnet, and dried at 70 °C. The final product was crushed to obtain the Fe_3_O_4_ powder.

The MCM-41@Fe_3_O_4_ was prepared by the following method [31]: 2.74 g of cetyltrimethylammonium bromide (CTAB) was dissolved in 567 mL of deionized water. MNPs were dispersed in the solution, and the pH of the solution was adjusted to 11.0 with an ammonia solution. Then, 12.5 mL of tetraethylorthosilicate (TEOS) was slowly added to the solution within 10 min. The suspension was stirred for 2 h at room temperature and transferred to a hydrothermal reaction vessel for 18 h at 100 °C. The resulting black solid precipitate was then washed with deionized water until it was neutral, separated by a magnet, and dried at 90 °C for 36 h. Finally, the dried powder was gradually calcined at 550 °C for 4 h, and the samples obtained were denoted as MCM-41@ Fe_3_O_4_.

The MnO_2_/MCM-41@Fe_3_O_4_ samples were synthesized according to a procedure in the literature [20,32]. First, 1.5 g of KMnO_4_ and 0.275 g of MnSO_4_-H_2_O were dissolved in 40 mL of deionized water. Then, 0.943 g of MCM-41@ Fe_3_O_4_ was dispersed in the solution. Then, 1.5 g of KMnO_4_ was dissolved in 40 mL of deionized water and added to the suspension within 30 min. The mixture was then transferred to a hydrothermal reaction vessel and kept at 240 °C for 24 h. After cooling the reactor to room temperature, the sample was centrifuged/washed 5 times with deionized water to remove residual impurities. The solid was then dried at 105 °C for 24 h. Finally, the sample was calcined in a muffle furnace at 300 °C for 3 h and denoted as MnO_2_/MCM-41@Fe_3_O_4_.

### 2.3. Preparation of COFAC–FeMnMCM–ALG Composite Beads

A 1% (*w*/*v*) sodium alginate (ALG) solution was prepared by dissolving 0.1 g of the powdered ALG in 100 mL of deionized water, to which 0.1 g of MnO_2_/MCM-41@Fe_3_O_4_ and 0.2 g of COFAC were added, followed by stirring for 12 h. Then, 2% (*w*/*v*) CaCl_2_ was added using a burette to form hydrogel composite beads (denoted as COFAC–FeMnMCM–ALG). The beads were separated, washed with deionized water, and freeze-dried for 48 h. For comparison, the same procedure was used to prepare CaCl_2_ cross-linked ALG (0.1 g ALG and 100 mL H_2_O), COFAC/ALG (0.2 g COFAC, 0.1 g ALG and 100 mL H_2_O), FeMnMCM/ALG (0.1 g MnO_2_/MCM-41@Fe_3_O_4_, 0.1 g ALG and 100 mL H_2_O), and 2FeMnMCM/ALG (0.2 g MnO_2_/MCM-41@Fe_3_O_4_, 0.1 g ALG and 100 mL H_2_O) beads.

### 2.4. Characterization and Analytical Methods

SEM-EDS images of the composite beads were obtained using an FEI Nova Nano-SEM 450 (Hillsboro, OR, USA) and Zeiss GeminiSEM 300 (Oberkochen, Germany). The phase structural properties of the obtained materials were determined by XRD diffraction patterns in the range of 5° to 70° at a scan rate (2θ) of 2°/min using a Bruker D8 Advance Powder Diffractometer (Cu-Kα radiation) (Billerica, MA, USA). The metal coordination state with the composite beads was analyzed by XPS analysis using ThermoFisher K-Alpha equipment with Al-Kα radiation (hv = 1486.6 eV) (Waltham, MA, USA). FTIR results in the range of 4000–650 cm^−1^ were obtained using a Thermo-Fisher IS50. The TGA results were from a thermogravimetric analyzer (NETZSCH STA 449F5, Selb, Germany) under air atmosphere from 30 °C to 800 °C at a heating rate of 10 °C/min. The specific surface area, total pore volume, and average pore radius were determined by nitrogen adsorption at −196 °C (outgassing was performed at room temperature) using a Quantachrome Autosorb iQ gas sorption analyzer system (Boynton Beach, FL, USA). A LakeShore Company (Westerville, OH, USA) vibrating sample magnetometer (model 7404) was used to investigate the magnetic properties of the composite sample. A Bruker A300 spectrometer was used to study the reactive oxygen species (ROS) generated in the reaction system.

### 2.5. Catalytic TC Degradation Experiments

The as-prepared beads were then applied as catalysts for the catalytic degradation of tetracycline (TC). The initial concentration of the TC solution was 10 mg/L. The reaction mixture (total volume 30 mL, adjusted with H_2_O), consisting of 25 mL of TC solution and a dose of H_2_O_2_, was placed in a conical flask wrapped with aluminum foil to keep it in a dark condition. The catalysts (beads) were then immersed in 10 mL of deionized water for 10 min before being added to the mixture. The suspension was shaken at 180 rpm in a bath shaker covered with a black plastic sheet at 30 °C to perform the batch catalytic degradation experiment. Finally, 2.5 mL of the reaction mixture was taken from the suspension every 10 min, filtered, and measured using a UV-Vis spectrophotometer (Puxi TU1900, Beijing, China) under wavelength λ = 357 nm.

For the catalytic degradation reactions, a pseudo-first-order kinetic model was presented with respect to the reaction time. The kinetics of TC degradation are described by the following equation:

(1) $$-{\ln(\;C}_{t}{/C}_{0})=k_{obs}t$$

where C_0_ (mg/L) is the initial TC concentration in the solution, C_t_ (mg/L) is the TC concentration at time t, and *k*_obs_ is the rate constant (min^−1^).

### 2.6. Catalyst Reuse

After a TC catalytic degradation experiment was completed, the COFAC–FeMnMCM–ALG composite beads were recovered by using a magnet and cleaned with deionized water 3 times. The recovered composite beads (without drying) were then used again for the next round of the experiments. The catalyst was used 3 times by following the same steps/procedures outlined above.

## 3. Results and Discussion

### 3.1. Characterization of Catalysts

**Figure 1:**
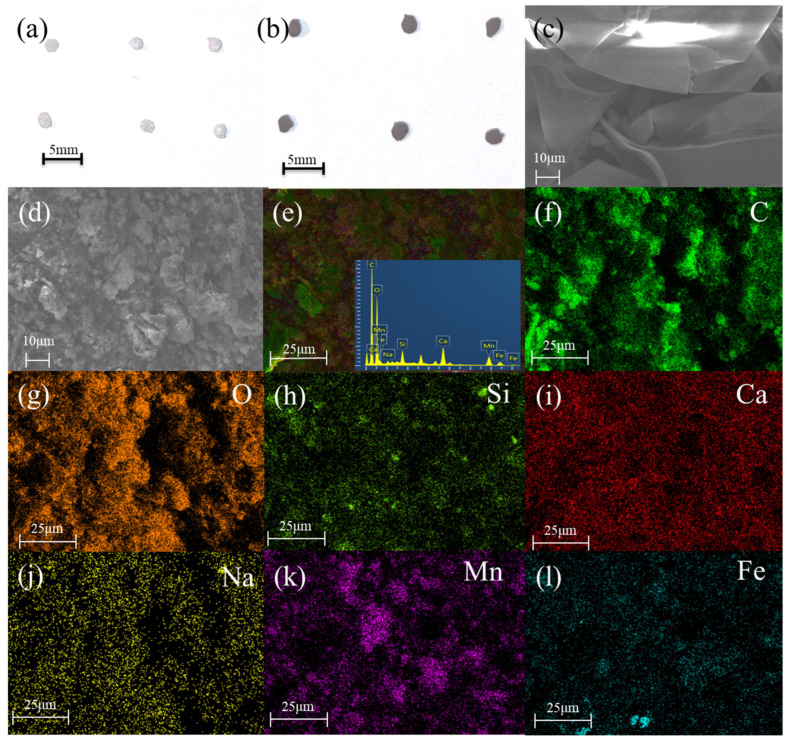
Optical pictures of (**a**) ALG-2%CaCl, and (**b**) COFAC–FeMnMCM–ALG composite beads; SEM image of cross section of (**c**) ALG-2%CaCl_2_ and (**d**) COFAC–FeMnMCM–ALG composite beads; EDS patterns of cross section of (**e**–**l**) COFAC–FeMnMCM–ALG composite beads.

All the beads are in their homogeneous spherical shape. The optical images of the ALG-2% CaCl_2_ beads and the COFAC–FeMnMCM–ALG composite beads (Figure 1a,b) showed that both beads had retained their shape and diameter after freeze drying, with a diameter of approximately 1 mm. The composite beads have a more irregular shape with a heterogeneous surface and a rougher surface, which is favorable for TC adsorption and catalysis because of the increased surface area [5,7,9].

The SEM results (Figure 1c) revealed that the ALG-2% CaCl_2_ beads had the gaps between the leaf-like structures and did not have many pores in their cross section. As shown in Figure 1d, the original structure was disrupted, and the gaps between pore walls in the COFAC–FeMnMCM–ALG composite beads were more tightly packed together. A porous structure with different diameters was exhibited [10,11], resulting in a larger specific surface area and pore volume. This could be demonstrated by the assembly of MCM-41 and COFAC, which created a new heterogeneous, rough, and interconnected filamentous structure [12] in the composite beads. This was probably due to the irregular growth of ice crystals during the freeze-drying process.

From the EDS results (Figure 1e–l) of the composite beads, the elemental contents of C, O, and Ca were 37.84%, 44.21%, and 2.56%, respectively. The presence of Mn (3.10%), Fe (1.08%), Si (1.19%), and Na (0.35%) in the cross-linked COFAC–FeMnMCM–ALG beads indicated the successful incorporation of MnO_2_/MCM-41 @Fe_3_O_4_, COFAC, and ALG, respectively. The SEM-EDS results confirmed that the COFAC–FeMnMCM–ALG aerogel composites were successfully prepared.

**Figure 2:**
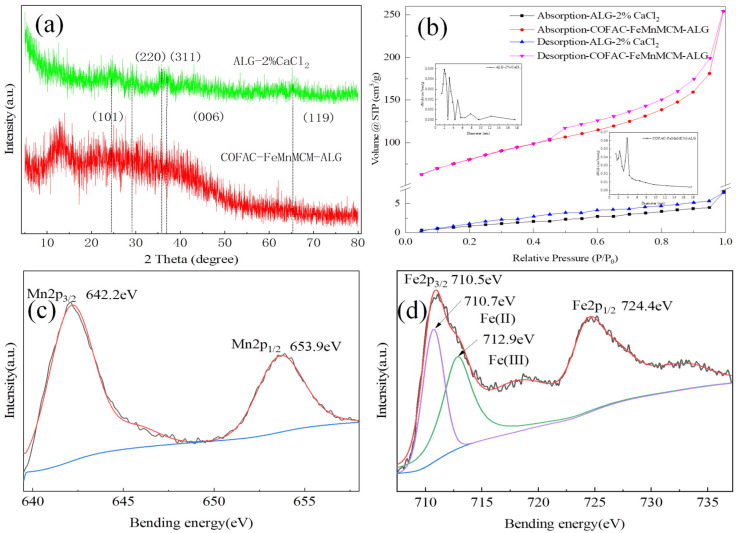
(**a**) XRD patterns and (**b**) N_2_ adsorption and desorption isotherms and pore-size distribution diagram for ALG-2%CaCl_2_ and COFAC–FeMnMCM–ALG composite beads; (**c**,**d**) XPS analysis of COFAC–FeMnMCM–ALG.

The samples of ALG-2% CaCl_2_ and COFAC–FeMnMCM–ALG beads were further characterized by XRD analysis, as shown in Figure 2a. The prepared ALG-2%CaCl_2_ beads had no intensive diffraction peaks, indicating that the sample had a predominantly amorphous structure [33,34]. The COFAC–FeMnMCM–ALG sample showed 2θ values at 24.9°, 36.8°, and 65.8°, which were assigned to the network plans (101), (006), and (119), respectively. These 2θ values show the same pattern as the standard XRD pattern of δ-MnO_2_ (JCPDS No. 18-0802) [35]. Also, the 2θ values at 35.7° (311) in the XRD pattern belong to Fe_3_O_4_ (JCPDS No. 89-0950) [36,37], which was consistent with a ferromagnetic property. The COFAC–FeMnMCM–ALG composite sample showed a simple overlap of the diffraction peaks of δ-MnO_2_ and Fe_3_O_4_, indicating that no new crystalline structures formed in the composite beads. The results indicated that the target catalysts had been successfully synthesized.

The N_2_ adsorption and desorption isotherms and pore size distribution (BJH model) of the ALG-2%CaCl_2_ and COFAC–FeMnMCM–ALG composite beads are shown in Figure 2b. According to IUPAC, the isotherms were categorized as type IV, indicating that the composite beads have a mesoporous structure [38]. The type H3 adsorption hysteresis loop indicated that the pores of the composite beads are mainly mesoporous and slit pores [39].

The COFAC had a large BET surface area of 1585.6 0 m^2^/g (Table 1) and a mesoporous structure with a total pore volume (V_T_) of 1.055 cm^3^/g. The S_BET_ and V_T_ of the MnO_2_/MCM-41@Fe_3_O_4_ sample were 81.34 m^2^/g and 0.6256 cm^3^/g, respectively.

The ALG-2% CaCl_2_ sample showed a low porosity of 0.011 cm^3^/g and a specific surface area of 6.46 m^2^/g. The COFAC–FeMnMCM–ALG sample showed a rough and interconnected filamentous structure, and the pores were partially and/or fully covered with COFAC and MnO_2_/MCM-41@Fe_3_O_4_, with an S_BET_ of 280.154 m^2^/g and a V_T_ of 0.3941 cm^3^/g, respectively. The D_p_ of the COFAC–FeMnMCM–ALG sample (3.826 nm, calculated using the BJH method) was larger than the molecular diameter of TC (1.27 nm) [40]. Thus, the TC molecules freely diffused in the spaces inside/outside the composite beads to accelerate its enrichment and catalytic degradation [26,41].

An XPS analysis was carried out on the COFAC–FeMnMCM–ALG composite bead sample (Figure 2c). The two peaks at 642.2 and 653.9 eV are assigned to the Mn 2p_3/2_ and Mn 2p_1/2_ peaks of MnO_2_, respectively [42]. Its high-resolution Fe 2p XPS spectrum was shown in Figure 2d, where the two peaks at 710.5 and 724.4 eV correspond to the Fe 2p_3/2_ and Fe 2p_1/2_ peaks of Fe_3_O_4_, respectively [43]. For Fe 2p_3/2_, the binding energies of 711.1 eV belonged to Fe(II), and the peaks at 713.5 eV were attributed to F(III) [44].

**Figure 3:**
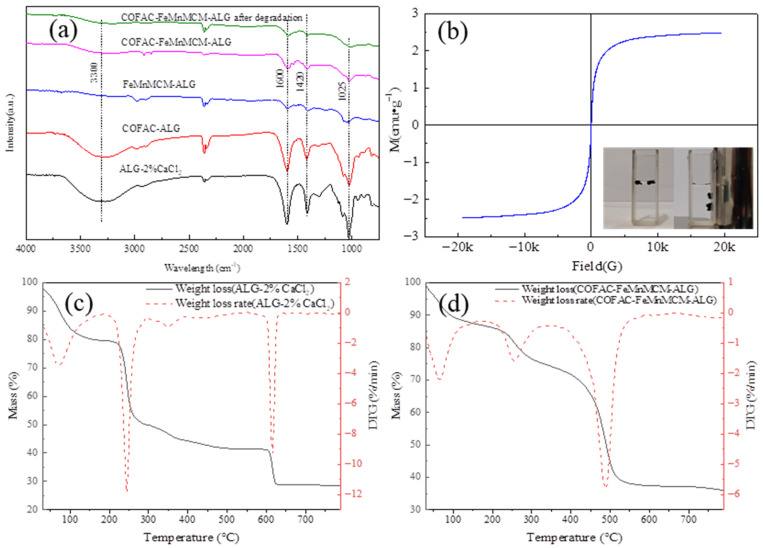
(**a**) FTIR spectra of beads: ALG-2%CaCl_2_, COFAC-ALG, FeMnMCM-ALG, COFAC–FeMnMCM–ALG, and COFAC–FeMnMCM–ALG after degradation; (**b**) the magnetic property of COFAC–FeMnMCM–ALG composite beads; (**c**,**d**) TG-DTG diagram of ALG-2%CaCl_2_ beads and COFAC–FeMnMCM–ALG composite beads.

The FTIR spectra of the ALG-2%CaCl_2_, COFAC-ALG, FeMnMCM-ALG, COFAC–FeMnMCM–ALG, and COFAC–FeMnMCM–ALG samples after degradation are shown in Figure 3a. A broad band corresponding to the stretching of the -OH groups present in the alginate polymer chain was observed between 3100 and 3640 cm^−1^. Weak bands at 1600 and 1420 cm^−1^ were attributed to the stretching of -COO^−^, most of which was bound to Ca^2+^ to form three-dimensional cross-linked networks [45]. The band at 1025 cm^−1^ was assigned to the vibrations of the C-O bond [26,46,47]. The interactions between -COO- (from ALG) and -OH (from COFAC) caused a slight shift of the characteristic bands of the carboxyl group (1600 and 1420 cm^−1^).

The magnetization curves of the COFAC–FeMnMCM–ALG sample were tested using a vibrating sample magnetometer (VSM), and the results are shown in Figure 3b. There were hardly any hysteresis loops on the magnetization curves, indicating that the composite beads exhibited superparamagnetic behavior. This property allowed for the easy recovery of used samples using an external magnet, as shown in Figure 3b. In addition, the saturation magnetization of the composite beads was 2.48 emu/g. This allowed the composite beads to be easily magnetically separated in water.

The TGA results (under air flow) of the ALG-2%CaCl_2_ beads and COFAC–FeMnMCM–ALG composite beads were obtained in the temperature range 30–800 °C (Figure 3c,d). For the ALG-2%CaCl_2_ beads shown in Figure 3c, the first stage of degradation was due to the water desorption at 30–200 °C. About 20.32% free water/bound water loss was recorded in this temperature range. This also showed that the composite beads had a good hydrophilic character, mainly due to interactions between water and the surface hydroxyl groups. The second degradation step occurred at temperatures higher than 200 °C, which was mainly due to the decomposition of the carbon chains and the formation of calcium carbonate (CaCO_3_)/sodium carbonate (Na_2_CO_3_) [48]. A mass loss of 38.12% occurred in this temperature range (200–350 °C). The last stage occurred in the range of 550–800 °C and was associated with the decomposition of the CaCO_3_/Na_2_CO_3_, with a weight loss of 13.09%. The TG residue was approximately 28.47%.

For the TGA analysis of the COFAC–FeMnMCM–ALG composite beads (Figure 3d), the same three thermal events were evident. The first stage occurred in the temperature range of 30–185 °C, with a weight loss of 13.01%. The second stage, with a weight loss of 13.19%, occurred in the range of 185 to 360 °C, which was mainly due to the decomposition of the carbon materials. The third stage, with a weight loss of 37.86%, occurred in the range of 360–800 °C and was due to the decomposition of CaCO_3_/Na_2_CO_3_ and the burning of activated carbon. The residue was about 35.93%. It could be observed that the moisture content of the pure ALG-2%CaCl_2_ beads was higher than that of the COFAC–FeMnMCM–ALG composite beads. This meant that the composites absorbed less water. The addition of activated carbon reduced the polarity and hygroscopicity of the alginate materials [49].

### 3.2. Effectiveness of As-Prepared Catalysts for TC Removal

**Figure 4:**
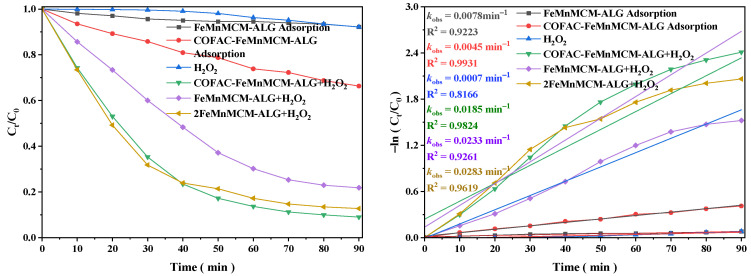
TC removal efficiency by different composite beads.

Adsorption and catalytic properties are critical for the destruction of TC in a catalytic degradation process [16,50,51]. The adsorption behavior of TC on the FeMnMCM-ALG and COFAC–FeMnMCM–ALG samples was shown in Figure 4. The removal efficiencies of TC by the COFAC–FeMnMCM–ALG beads without the presence of H_2_O_2_ were higher than those obtained by the FeMnMCM-ALG beads. Specifically, about 33.7% and 7.8% of the TC were removed in 90 min, respectively. This indicated that the addition of COFAC could effectively reinforce the adsorption performance of the composite beads for TC. When H_2_O_2_ was used alone without a catalyst, the removal efficiency of TC was about 7.8% in 90 min due to the spontaneous self-decomposition of H_2_O_2_, which produces ROS for TC degradation. When COFAC–FeMnMCM–ALG and H_2_O_2_ were added together, the removal efficiency of TC reached nearly 91.0% within 90 min, and the *k*_obs_ was 0.0283 min^−1^, which were much higher than those when using COFAC–FeMnMCM–ALG as the adsorbent and H_2_O_2_ alone, indicating that the addition of a MnO_2_/MCM-41@Fe_3_O_4_ catalyst significantly promotes the activation of H_2_O_2_ to generate abundant ROS for TC degradation.

When FeMnMCM-ALG and 2FeMnMCM-ALG were also used as catalysts together with H_2_O_2_, the removal efficiencies of TC reached 78.2% and 87.3% within 90 min, and the *k*_obs_ values were 0.0185 min^−1^ and 0.0233 min^−1^, indicating that the MnO_2_/MCM-41@Fe_3_O_4_ beads play a very important role in the degradation of TC. The removal efficiencies of TC were lower than that of the COFAC–FeMnMCM–ALG sample. This was due to the presence of COFAC with a strong biosorption capacity and rich functional groups, allowing for the fast enrichment of TC into the interior structure [27], increasing the concentration of tetracycline next to the catalytic sites, thus accelerating the catalytic degradation rate of TC.

### 3.3. Effects of Process Parameters on TC Degradation

To study the effect of the H_2_O_2_ concentration on the TC degradation rate, various concentrations of H_2_O_2_ were used while keeping the other parameters constant. The concentration of H_2_O_2_ can affect the pH of the solution, which will determine the form in which TC exists. Depending on the solution’s pH, TC exits in three forms, such as a cationic form at pH < 3.3, zwitterionic at pH 3.3–7.7, and anionic at pH > 7.7 [52]. The pH of the solution was measured at the beginning and during the reaction. The initial concentration of H_2_O_2_ was set to 0%, 1%, 3%, 5%, and 7%, and the pH values were 5.68, 5.64, 5.60, 5.44, and 5.37, respectively. Taking the reaction of a 5% concentration of H_2_O_2_ as an example, we measured the pH values of the reaction solution after 30, 60, and 90 min. The recorded values were 6.46, 6.68, and 6.75, respectively. This demonstrates that the TC remains in a zwitterionic form throughout the reaction.

**Figure 5:**
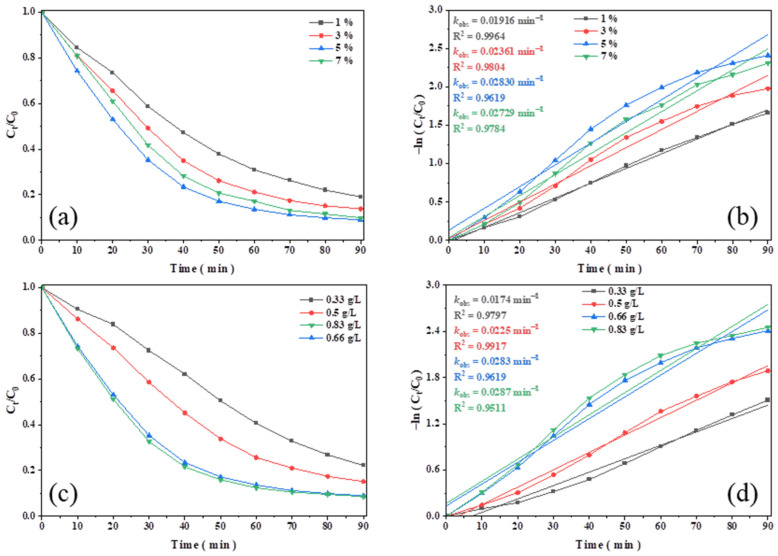
Effects on the degradation of TC: (**a**,**b**) initial concentrations of H_2_O_2_; (**c**,**d**) catalyst dosage.

Figure 5a,b demonstrate that as the initial concentration of H_2_O_2_ was increased from 1% to 5%, the degradation of TC increased from 72% to 91%, and the *k*_obs_ increased from 0.0192 to 0.0283. This indicated that a higher concentration of H_2_O_2_ promoted the formation of more ·OH [43,53,54]. However, the degradation rate of TC slightly decreased when the initial concentration of H_2_O_2_ was increased to 7%. The excessive amounts of H_2_O_2_ may have some side reactions with ·OH, such as those shown in Equations (2) and (3) [32,55,56]. The formation of either ·HO_2_ with a weak oxidizing capacity or O_2_ with no oxidizing properties can decrease the overall TC degradation rate [29,57].

(2) $$\cdot \mathrm{OH}\;+{\;H}_{2}O_{2}\;\to \;\cdot {\mathrm{HO}}_{2}+{\;H}_{2}O\;$$

(3) $$\cdot \mathrm{OH}\;+\cdot {\mathrm{HO}}_{2}\;\to \;\cdot {\mathrm{HO}}_{2}+{\;O}_{2}\;$$

Figure 5c,d show the effect of the catalyst dosage on the TC degradation performance. The degradation efficiency was increased from 77.8% to 91.0%, and *k*_obs_ increased from 0.0174 to 0.0283 min^−1^ when the catalyst amount increased from 0.33 g/L to 0.66 g/L. Catalysts with more active sites promote the production of more radicals (·OH and ·O_2_^−^) [29,57], which could improve the degradation rate. However, when the catalyst amount was increased to 0.83 g/L, the degradation efficiency and *k*_obs_ increased only slightly (to 91.4% and 0.0287). This could be due to the following reasons. Firstly, an excessive amount of catalyst increased the collision probability of catalyst particles in the solution, which obscured some of the active sites and reduced the degradation efficiency [32]; on the other hand, the excessive amount of catalyst could also reduce the mass transfer rate of H_2_O_2_ and TC in the solution, leading to a reduction in catalytic activity [43].

**Figure 6:**
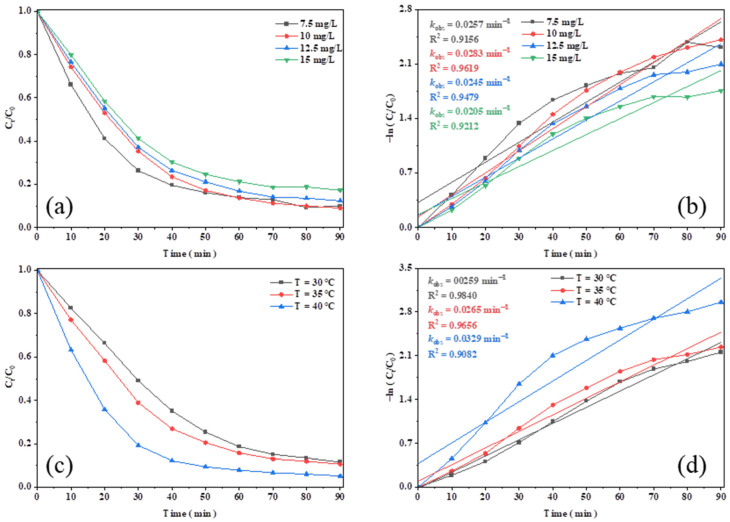
Effects on the degradation of TC: (**a**,**b**) initial TC concentration and (**c**,**d**) temperature.

The effect of the initial TC concentration was investigated, and the results are shown in Figure 6a,b. The degradation efficiency and rate of TC removal decreased with an increase in the initial TC concentration. This was because, on the one hand, the competitive adsorption of TC and H_2_O_2_ at the active site may decrease the formation of ·OH [35]. On the other hand, with the amounts of catalyst and H_2_O_2_ in the solution fixed, the increased pollutant loading means that a lower amount of ·OH would be available for a given number of pollutants [36].

The reaction temperature usually has a significant effect on the degradation efficiency and rate. Figure 6c,d show that the degradation rate of TC increased with the increase in temperature, indicating that more ·OH was produced [55].

### 3.4. Catalyst Reuse

**Figure 7:**
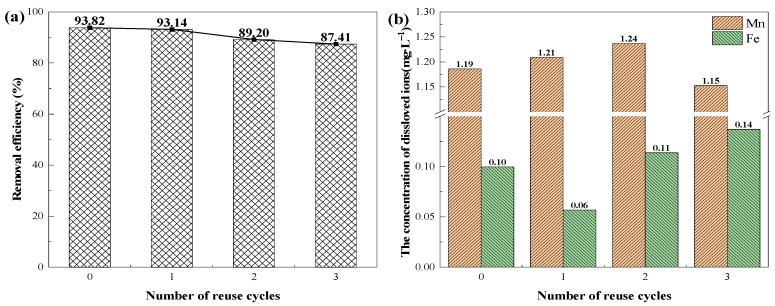
(**a**) Reuse of COFAC–FeMnMCM–ALG on the removal of TC. (**b**) Iron leaching during reuse of COFAC–FeMnMCM–ALG.

The reuse of the as-prepared catalysts was studied. The recovered composite beads were cleaned by rinsing with deionized water five times. This allowed them to be reused for the next cycle without drying out. Figure 7a indicates that the composite beads exhibited strong catalytic activity for three cycles, and the degradation efficiency of TC decreased only slightly, from 93.82% to 87.41%. This could be due to the depletion of the surfactant active site after multiple uses of the catalyst [27] or the blocking of the catalyst by the absorbed TC and TC oxidation products [58]. Nevertheless, a removal efficiency of 87.41% could still be achieved after a total of four recycling runs, suggesting a high degree of stability and reusability of the composite beads.

Ion leaching in the reuse experiment was shown in Figure 7b. The maximum leaching manganese ion concentration was 1.24 mg/L, and the maximum iron ion dissolution concentration was only 0.14 mg/L. This indicated that the composite beads were stable, which was consistent with the reuse experiments.

### 3.5. Identification of Hydroxyl Radical as the Main Reactive Species

**Figure 8:**
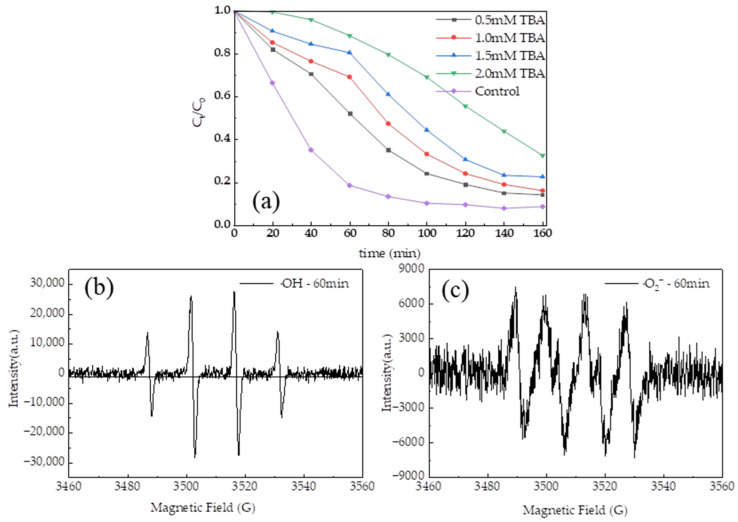
(**a**) Effects of TBA on TC degradation; EPR spectra of radicals with different radical scavengers: (**b**) DMPO-OH and (**c**) DMPO-O_2_^−^.

In a typical catalytic system using H_2_O_2_ and Fe_3_O_4_/MnO_2_ catalysts, ·OH radicals are usually the main free radicals involved in the reaction [59]. In the present study, tert-butanol (TBA) was used as the ·OH radical scavenger. The experimental results (Figure 8) showed that increasing the concentration of TBA leads to a decrease in the TC degradation rate. It was noteworthy that when 2.0 mM of TBA was added, the degradation of TC was minimal within the first 20 min, confirming that ·OH played a dominant role in this system. However, as the reaction progressed, TBA was continuously consumed and the concentration was continuously reduced; thus, the TC degradation gradually picked up. Furthermore, some other free radicals, including ·O_2_^−^, might have been generated in the system and could not be neutralized by TBA, which can also contribute to the TC degradation.

The types of free radicals in the system were identified using electron paramagnetic resonance spectroscopy (EPR), and the results are displayed in Figure 8b,c to further support the above conclusions. DMPO (5,5-dimethyl-1-pyrroline N-oxide) was used as a radical scavenger in the EPR studies, with a magnetic field varying between 3460 and 3560 Gauss (G). The experimental conditions were as follows: 10 mg of composite beads, 12.5 mL of TC solution, and 2.5 mL of H_2_O_2_. Samples were taken to identify ·OH free radicals in the water phase and ·O_2_^−^ free radicals in the methanol phase, respectively, after the reaction had been running for 60 min.

As can be seen from Figure 8b, when the catalysts were added to the system, four symmetrical peaks with a signal intensity ratio of approximately 1:2:2:1 were observed, which was a typical characteristic signal peak of ·OH. Figure 8c illustrates the generation of four symmetrical peaks with a signal intensity ratio of about 1:1:1:1, while producing two asymmetric peaks with weaker intensity. This was a typical characteristic signal peak of ·O_2_^−^. Furthermore, the signal strength of ·OH was three times stronger than that of ·O_2_^−^ under identical experimental conditions, suggesting that hydroxyl radicals were the main species in the solution.

### 3.6. Proposed Mechanism for the Catalytic Degradation of TC

In the system, ·OH and ·O_2_^−^ are formed from the catalytic reaction of Fe_3_O_4_ and MnO_2_ with H_2_O_2_ [60]. Mn^4+^ can accelerate the rapid conversion between Fe^3+^ and Fe^2+^ [61]. The presence of COFAC enriches the TC concentration in the catalyst due to its excellent adsorption capacity, which further accelerates the TC catalytic degradation process. By considering those available in the literature [60,61], we proposed the corresponding reaction mechanism, as shown in Equations (4)–(9).

(4) $${\mathrm{Fe}}^{2+}+{\;H}_{2}O_{2}\to {\;\mathrm{Fe}}^{3+}+\cdot \mathrm{OH}\;+{\;\mathrm{OH}}^{-}$$

(5) $${\mathrm{Fe}}^{3+}+{\;H}_{2}O_{2}\to {\;\mathrm{Fe}}^{2+}+\cdot {\mathrm{HO}}_{2}+{\;H}^{+}$$

(6) $${\mathrm{Mn}}^{4+}+{\;H}_{2}O_{2}\to {\;\mathrm{Mn}}^{3+}+\cdot {\mathrm{HO}}_{2}+{\;H}^{+}$$

(7) $${\mathrm{Mn}}^{3+}+{\;\mathrm{Fe}}^{3+}\to {\;\mathrm{Mn}}^{4+}+{\;\mathrm{Fe}}^{2+}$$

(8) $$\cdot {\mathrm{HO}}_{2}\;\to \;\cdot O_{2}^{-}+{\;H}^{+}$$

(9) $$\cdot \mathrm{OH}\;+TC\;\to {\;\text{Mid-product}+H}_{2}O\;$$

**Figure 9:**
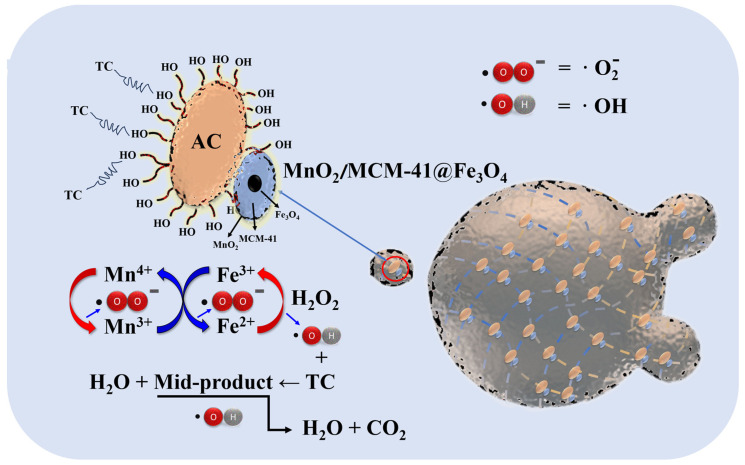
Proposed schematic diagram of H_2_O_2_-induced degradation of TC by the as-prepared COFAC–FeMnMCM–ALG composites catalysts.

Based on Equations (4)–(9), the catalytic degradation mechanism of TC by the COFAC–FeMnMCM–ALG composite beads was proposed (Figure 9).

## 4. Conclusions

Novel magnetic-active composite beads, COFAC–FeMnMCM–ALG, were prepared by first mixing MnO_2_/MCM-41@Fe_3_O_4_ (FeMnMCM) and COFAC (*Camellia oleifera* shell activated carbon) and then encapsulated using alginate (ALG) and CaCl_2_ as a cross-linking matrix. The as-prepared composite beads were comprehensively characterized by various techniques and were subsequently applied as catalysts in the H_2_O_2_ oxidative degradation of tetracycline (TC). The MnO_2_/MCM-41@Fe_3_O_4_ demonstrated excellent catalytic capacity, while the COFAC had strong bioadsorption capacity for TC, which facilitated the catalytic oxidative degradation process. The electron paramagnetic resonance (EPR) results showed that both ·OH and ·O_2_^−^ radical species were generated in the system, and the quenching experiments supported the notion that ·OH was the main radical species. Furthermore, the catalytic degradation experiments support the conclusion that the as-prepared COFAC–FeMnMCM–ALG composite catalysts had a strong catalytic performance and adsorption capacity during the catalytic degradation of TC and were readily recycled or reused. This study presents a novel, easily separable, and recyclable composite catalyst that is suited to AOPs (advanced oxidation processes).

## Figures and Tables

**Table 1:** Structure parameters of ALG-2%CaCl_2_ beads, COFAC and MnO_2_/MCM-41@Fe_3_O_4_ and COFAC–FeMnMCM–ALG composite beads.

Samples	BET Surface Area (m^2^/g)	Total Pore Volume (cm^3^/g)	Pore Diameter (nm)
ALG-2%CaCl_2_	6.458	0.01093	1.938
COFAC	1585.602	1.055	3.930
MnO_2_/MCM-41@Fe_3_O_4_	81.338	0.6256	17.522
COFAC–FeMnMCM–ALG	280.154	0.3941	3.826

## Data Availability

Data are contained within the article.
